# Introduced populations of ragweed show as much evolutionary potential as native populations

**DOI:** 10.1111/eva.13211

**Published:** 2021-04-02

**Authors:** Brechann V. McGoey, John R. Stinchcombe

**Affiliations:** ^1^ Ecology and Evolutionary Biology Department University of Toronto Toronto ON Canada; ^2^ Koffler Scientific Reserve University of Toronto Toronto ON Canada

**Keywords:** additive genetic variance, *Ambrosia artemisiifolia*, G matrices, introduced species

## Abstract

Invasive species are a global economic and ecological problem. They also offer an opportunity to understand evolutionary processes in a colonizing context. The impacts of evolutionary factors, such as genetic variation, on the invasion process are increasingly appreciated, but there remain gaps in the empirical literature. The adaptive potential of populations can be quantified using genetic variance–covariance matrices **(G)**, which encapsulate the heritable genetic variance in a population. Here, we use a multivariate Bayesian approach to assess the adaptive potential of invasive populations of ragweed (*Ambrosia artemisiifolia*), a serious allergen and agricultural weed. We compared several aspects of genetic architecture and the structure of **G** matrices between three native and three introduced populations, based on phenotypic data collected in a field common garden experiment. We found moderate differences in the quantitative genetic architecture among populations, but we did not find that introduced populations suffer from a limited adaptive potential or increased genetic constraint compared with native populations. Ragweed has an annual life history, is an obligate outcrosser, and produces very large numbers of seeds and pollen grains. These characteristics, combined with the significant additive genetic variance documented here, suggest ragweed will be able to respond quickly to selection pressures in both its native and introduced ranges.

## 1 INTRODUCTION

Anthropogenic influences, from climate change, to habitat destruction, to pollution, are altering ecosystem function and diminishing native biodiversity across the globe. One important way humans are changing the ecological landscape is through the accidental or intentional movement of organisms into novel locations. The ecological and economic impacts of alien plants continue to be immense (Sakai et al., 2001), and for this reason, it is important for both applied and basic research reasons to understand how and why certain plant populations become invasive. Whether and how invasive species evolve in their new range is key to understanding their establishment and success (Colautti & Barrett, 2013), yet we have a weak understanding of the evolutionary potential of size, performance, and life history traits in introduced species and their role in invasion success. Here, we use a quantitative genetic approach to compare multivariate evolutionary potential between introduced and native populations of the prolific invader *Ambrosia artemisiifolia* (common ragweed).

Most research on species invasions has focused on possible ecological explanations and consequences, while the evolutionary determinants and outcomes have only recently been emphasized, and remain less well understood (Bacigalupe, 2009). Recent evidence suggests that evolutionary responses can be more important in determining invasion success and spread than traditional ecological explanations. For example, local adaptation accounted for greater fitness effects than enemy release and allocation to competitive ability (EICA) in a study of *Lythrum salicaria* (Colautti & Barrett, 2013). Whereas there has long been an emphasis on individual‐level traits, it is increasingly recognized that population‐level factors such as genetic variation will have major impacts on the ability of a species to establish in a new environment and to respond to natural selection (Bacigalupe, 2009; Crawford & Whitney, 2010). Adaptation can be a pivotal factor in allowing a colonizing population to establish and spread (Colautti & Barrett, 2013; Huey et al., 2005). Since almost all traits that are likely to be under selection in a new environment are quantitative (Dlugosch & Parker, 2008a), characterizing the quantitative genetic variation of invasive populations is critical for understanding invasion success.

Despite the importance of heritable genetic variation for the ability of a population to respond to selection, there is a dearth of studies on invasive species from a quantitative genetics perspective (Bacigalupe, 2009) and neither theory nor logic offer straightforward *a priori* predictions. While there is often an assumption that all introduced populations will suffer from founder effects, any initial bottlenecks can be mitigated by multiple introductions (Roman & Darling, 2007); postintroduction hybridization or admixture can also create novel genotypes not found in the native range, fueling adaptation and invasion (see Bock et al., 2015; Dlugosch & Parker, 2008a, 2008b). Patterns of neutral genetic variation are unlikely to be helpful, as they are often uncorrelated with heritable quantitative variation (Mittell et al., 2015; Reed & Frankham, 2001). The presence and magnitude of epistasis effects (Monnahan & Kelly, 2015), and linkage disequilibrium (Hill & Maki‐Tanila, 2015) both impact additive genetic variance, and neither can be captured by simple measures of neutral genetic variation. Quantitative variation will be less impacted by losses of rare alleles (Dlugosch & Parker, 2008a), and some theory and empirical studies suggest epistatic and dominance variance can actually occasionally be converted to additive variance (Bryant et al., 1986; Cheverud & Routman, 1996; Goodnight, 1988; Willis & Orr, 1993, but see Barton & Turelli, 2004; Turelli & Barton, 2006). Invasive species are also often subject to strong directional selection, which in principle could create negative linkage disequilibrium among alleles affecting the selected traits, potentially reducing the amount of additive genetic variance (i.e., due to the Bulmer effect). Many studies of introduced species also suffer from insufficient sampling replication within the introduced and native ranges (Colautti et al., 2009). Collectively, these diverse evolutionary genetic and ecological processes that can affect quantitative genetic variation defy simple predictions for invasive species: Quantitative genetic variance may increase, decrease, or be unaffected by the processes leading to invasion. Ultimately, understanding an invasive species’ capacity to respond to selection and evolve, and whether that differs from its native range, is fundamentally an empirical question that requires directly comparing additive genetic variation (*V*_A_) and covariation (**G**) in multiple introduced and native populations, given the many potential evolutionary genetic mechanisms that could potentially affect genetic variance and covariance in introduced populations.

There have been numerous calls in the literature for quantitative genetic comparisons between native and invasive populations (e.g., Bacigalupe, 2009; Dlugosch & Parker, 2008a, 2008b; Lawson Handley et al., 2011), and the role of quantitative genetic variance in invasion biology has been long recognized (e.g., Lewontin, 1965). The presence of postinvasion local adaptation (e.g., Colautti & Barrett, 2013; see Bock et al., 2015 for other examples), which requires quantitative genetic variance in traits and fitness, suggests that such variance is not eliminated by the invasion process. Despite this, we lack an overall consensus about whether invasive species have increased, decreased, or unaffected genetic variance in the traits likely to be important for invasion success (e.g., life history, phenology, size, and other fitness‐related traits), and thus whether postinvasion evolutionary responses are likely to be reduced, unaffected, or even accelerated. Characterizing quantitative genetic variation in invasive populations is necessary for understanding whether or how species will evolve in a new range, and their potential for invasion success (Colautti & Barrett, 2013).

A well‐established literature on variation in single traits has uncovered genetic variance in almost all of them (Lynch & Walsh, 1998), which can falsely lead to the assumption that limited genetic variance is not a significant barrier to adaptive evolution (Blows & Hoffmann, 2005). Even if there is additive genetic variance for a univariate trait, there can still be genetic constraints on adaptation due to covariances with other traits (Agrawal & Stinchcombe, 2009; Lande & Arnold, 1983; McGuigan, 2006). The ability of a population to respond to a selective force will be dictated by the available variance in multiple traits along with the covariances between those traits. These genetic variances and covariances are summarized by the genetic covariance matrix, **G**; recall that genetic covariances are due to linkage disequilibrium between loci, or loci having pleiotropic effects on multiple traits, while phenotypic (co)variances reflect both genetic and environmental influences on traits (Lynch & Walsh, 1998). A multivariate genetic framework, incorporating the impacts of multiple traits and their genetic correlations, is necessary for a comprehensive understanding of the available genetic variation in a population (Blows, 2007; Blows & Hoffmann, 2005; Walsh & Blows, 2009).

The **G** matrix summarizes the available genetic variances and covariances and offers an integrated view of quantitative genetic variation, which allows for the estimation of constraints (Blows, 2007; Lande, 1979). Since it includes limitations caused by relationships among traits, it can expose constraints on adaptation, even in cases where all the traits individually have sufficient genetic variation (Blows, 2007; Dickerson, 1955). The **G** matrix will dictate the speed and direction of a population's response to selection (Steppan et al., 2002). Understanding evolutionary potential in invasive species therefore requires a comparison of **G** matrices between invasive and native populations to determine whether invasive species face genetic constraints and will have reduced phenotypic evolutionary potential. In addition to the uncertain effects of introductions on quantitative genetic variance described above, traditional evolutionary forces such as drift, migration, and selection can in principle affect **G** (e.g., Guillaume & Whitlock, 2007; Jones et al., 2003; Phillips et al., 2001; Turelli, 1988; also see Colautti & Barrett, 2011). Meta‐analyses also suggest that the environment can affect **G** directly (i.e., through G*E) in ways comparable to evolved differences between populations (Wood & Brodie, 2015). Invasive species are likely to experience all of these evolutionary and genetic forces simultaneously that can in principle affect **G**: bottlenecks and potentially enhanced drift, multiple introductions, novel environments, and altered patterns of migration and selection. It remains an open question whether and how these forces affect **G** in natural populations of invasive species in ways suggested as possible by theory and laboratory studies.

In this study, we examine how quantitative genetic architecture varies between introduced and native populations of the prolific invader *Ambrosia artemisiifolia* (common ragweed). We focus on size and phenology traits that are likely to be under selection in the native and introduced ranges, and likely to lead to continued invasion success, agricultural problems, and allergenic symptoms. Specifically, we ask the following questions: (1) How do native and introduced populations differ in their mean phenotypes, genetic variances, and heritabilities in key phenotypic traits? (2) Are there correlations between traits that could accelerate or constrain adaptation? (3) Is there a divergence in the **G** matrices of native and introduced populations? (4) How would native and invasive populations differ in their responses to selection based on their genetic (co)variances?

## 2 METHODS

### 2.1 Study species

*Ambrosia artemisiifolia* is an annual herb (Bassett & Crompton, 1975) and is self‐incompatible (Friedman & Barrett, 2008; Li et al., 2012). Preferring open habitats, it occurs in disturbed areas and is a common agricultural weed (Bassett & Crompton, 1975). A native to North America, *A*.* artemisiifolia* has spread to Europe, Asia, South America, and Australia (Friedman & Barrett, 2008). Ragweed produces around 1.2 billion grains of pollen per individual (Fumanal et al., 2007). During its flowering season in the late summer and early fall, it is the major cause of hay fever (Bassett et al., 1976), and about 10% of people test positive for allergies to *Ambrosia* (Gergen et al., 1987). In Europe, it is both a public health concern and the cause of crop yield losses (Fenesi & Botta‐Dukát, 2012). Unsurprisingly, given that ragweed is a wind‐pollinated obligate outcrosser, *F*_ST_ values are low (mean *F*_ST_ = 0.025, range = −0.019, 0.096, Martin, 2011, while McGoey et al., 2020 estimated *F*_ST_ = 0.056 for European samples and *F*_ST_ = 0.06 for North American samples). Phenotypic diversity and neutral marker diversity are high in the invasive range, suggesting evidence of multiple introductions (Genton et al., 2005; van Boohemen et al., 2017).

### 2.2 Seed collection and preparation

**FIGURE 1:**
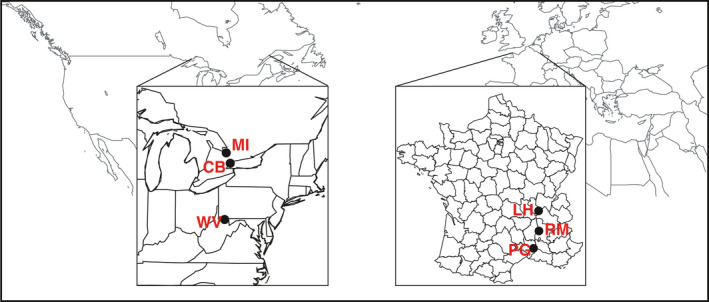
Map of *Ambrosia artemisiifolia* collection sites. Seeds were collected from at least 200 plants in each population in the fall of 2012. Note that the North American samples span latitudes 39.66−44.38 °N, while the French samples span 43.95–45.66 °N

We collected seed from three populations from both the native (Canada and the United States) and introduced (France) ranges (Figure 1), from overlapping latitudinal ranges (North America: 39.6584–44.37683 °N, France: 43.9475–45.66117 °N) (Colautti et al., 2009). All the populations were large, ranging from hundreds to tens of thousands of individuals. We sampled seed from at least 200 plants in each population. Sampling was haphazard, but we avoided collecting from plants adjacent to each other. Using methods adapted from Willemsen (1975) and Friedman (personal communication), we stratified seeds at 4°C for three months in plastic bags filled with silica and distilled water.

### 2.3 Parental generation

Beginning in January 2013, we removed seeds from stratification (4°C) and placed them on filter paper in petri dishes. We placed all the petri dishes in the greenhouse and monitored them for germination and signs of desiccation. As seeds germinated, we planted them into a 75% Pro‐Mix, 20% sand, and 5% topsoil soil mix in seedling flats. Petri dishes and early germinants were moistened and watered as needed, and all dishes and plants were treated equally. After four weeks, we transplanted the plants into 4‐inch round pots. To keep plants small and accelerate time to flowering, we compressed the growing season by switching the lights from long‐day photoperiods (14:10) to short‐day photoperiods (10:14), mimicking natural changes in daylength experienced after the summer solstice. To prevent uncontrolled pollination, we used individual chambers and a purified air delivery system (McGoey et al., 2017). Each plant was placed in a chamber in advance of flowering. The chambers were composed of plastic bags attached to Styrofoam rings, which fit tightly around each pot. The plastic bags were inflated with purified air, and individual plants were only removed from the grid for controlled crosses (see McGoey et al., 2017 for more details). All individuals in the parental generation experienced the same common environment (soil mix, pots, light, and purified air chambers).

### 2.4 Crossing design

Breeding designs are critical to partition components of variance in traits (Conner & Hartl, 2004; Falconer & Mackay, 1996; Lynch & Walsh, 1998). We used a nested paternal half‐sibling design, where we crossed a group of sires (pollen donors) to multiple randomly chosen dams (pollen recipients) (Falconer & Mackay, 1996). For each population, we had 50 sires crossed to three unique dams each (150 crosses per population) for a total of 900 unique crosses. Individual sires were used only with their three unique dams, and no sires or dams were re‐used.

### 2.5 Offspring generation

The offspring generation was grown at the Koffler Scientific Reserve (www.ksr.utoronto.ca; 44.803°N, 79.829°W) during the summer of 2014. As with the parental generation, we stratified seeds and then placed them on petri dishes to germinate. We transplanted seedlings from flat trays four weeks after germination into three blocks. Prior to planting, we removed all vegetation and tilled the soil. At the end of June, seedlings were transplanted over three days into the field. Within each block, we arranged plants in square grids with 10‐cm spacing between plants in a grid. We transplanted 2700 germinants (= 9 per paternal half‐sibling family) into the field. Failed establishment and early mortality reduced sample sizes, although the median number of paternal half‐sibling families per trait ranged from 39 to 49 (sample sizes of sires, dams, and total plants per paternal half‐sibling family are given in Table S2). To promote establishment, we supplemented with water and removed interspecific competitors within the plots for three weeks after transplantation. We also removed interspecific competition adjacent to the plots to prevent shading.

We measured early height (at two weeks), final height, final number of branches, and date of first flower. We observed little variation in germination timing, suggesting that most variation in early height was due to establishment and growth over the first two weeks, although subtle differences in germination timing might lead to contributions of plant age in early height. Final height was measured in early October, after vertical growth appeared to have stopped, and prior to a killing frost, which would have made estimates of reproductive effort impossible. Most ragweed plants are monoecious (Bassett & Crompton, 1975), and so we measured proxies of both male and female fitness. We used the total inflorescence length, which is correlated with pollen production (Fumanal et al., 2007), as a proxy for male reproductive effort. For female reproductive output, we used seed mass that is highly correlated with seed number (*r* ^2^ = 0.96, *p* < 0.001) (MacDonald & Kotanen, 2010).

### 2.6 Statistical analyses

All statistical analyses were conducted in R version 3.3.1 (R Development Core Team, 2016).

#### 2.6.1 Differences in means

We first performed a MANOVA to examine whether there was multivariate divergence between continents and populations in mean phenotypes. We used all six traits (early height, final height, branch number, time to first flower, male fitness, and female fitness) as response variables. Independent variables included the fixed effects of continent, and population nested within continent.

### 2.7 Bayesian quantitative genetic analysis

Historically, it has been difficult to estimate uncertainty around quantitative genetic parameters (Morrissey et al., 2014), but Bayesian Markov chain Monte Carlo (MCMC) methods offer a feasible solution (Hadfield, 2015). Using a Bayesian MCMC framework enabled us to include uncertainty when estimating the genetic variances and covariances in each population and then carrying those forward through all subsequent analyses and comparisons among populations (Aguirre et al., 2014; Teplitsky et al., 2014). Specifically, we sampled from the posterior distribution of the estimated parameters multiple times to characterize uncertainty in the estimated quantitative genetic parameters. Posterior distributions remain valid for downstream calculations and algebraic operations (Hadfield, 2010; Wilson et al., 2010; 2011), and by performing operations (matrix multiplication, estimation of heritability, etc) on many samples of the posterior, it is possible to characterize uncertainty in the calculated metrics.

Estimates of variance components will be constrained to values greater than zero (Walter et al., 2018), which poses challenges for traditional statistical significance testing. To assess the significance of our estimates, we generated a null distribution based on permuting phenotypes within populations. We first created 1000 randomized datasets for each population, where trait values were sampled without replacement and randomly assigned to sires and dams. We then fit the models exactly as described below, but on permuted data, for all 1000 randomized datasets to generate a distribution of expected outcomes under the null. We used these null expectations in both subsequent univariate and multivariate analyses to assess the significance of estimated variance components—in essence, asking whether the observed phenotypic similarity among related individuals in the experiment was greater than would be expected by random chance.

#### 2.7.1 Univariate analyses

We started by fitting a univariate model for each response variable, partitioning variation among sires (*V*_S_) and dams within sires (*V*_D_), separately for each geographic population. We used a burn‐in of 200,000 iterations, ran the model for 5,000,000 iterations, and sampled the posterior every 500 iterations to obtain 10,000 samples of the posterior distribution. We monitored autocorrelations between samples and convergence using the coda suite of tools (Plummer et al., 2006). Preliminary inspection of variance components did not show evidence of Markov chains becoming “stuck” near zero, so we did not pursue parameter‐expanded priors (cf Puentes et al., 2016). From the estimated sire and dam variance components (see Table S1), and the residual variance (*V*_E_), we estimated the narrow‐sense heritability for each trait within each population.

Since we used a half‐sibling breeding design, additive genetic variance (*V*_A_) was four times the variation in half‐sibling families within a population (*V*_S_) (Lynch & Walsh, 1998); note that when $\frac{V_{S}}{V_{s}+V_{D}+V_{e}}>0.25$, estimated narrow‐sense heritability values will be >1, which has been observed before (Hill & Thompson, 1978). Heritability values in Table S1 are narrow‐sense estimates of *V*_A_/*V*_P_. To assess whether observed heritabilities were greater than expected by chance, we compared them with the 95% HPD intervals of the randomized heritability estimates.

#### 2.7.2 Estimation of G matrices

We also used *MCMCglmm* to generate **G** matrix estimates for the six traits. We explored a variety of different priors for the expectations of variances and degrees of belief in MCMC models, ranging from diagonal matrices, sire and dam covariance matrices equaling ½ the phenotypic covariance matrix (**P**), and sire and dam covariance matrices equaling 1/4**P**. We compared the output of different prior specifications with the Gelman and Rubin diagnostic (Plummer et al., 2006) and found that they sampled the same posterior distribution (Gelman & Rubin, 1992; Puentes et al., 2016). In the results below, we present output from models with priors where the expectations of sire and dam covariance matrices were equal to 1/4**P**, with degree of belief (nu = *n* − 0.998, where *n* is the number of traits). We used 500,000 burn‐in iterations and 5,000,000 total iterations, and sampled the posterior every 500 iterations to obtain 10,000 samples of the posterior distribution of **G**.

In these models, block was treated as a fixed effect and dam was nested within sire; we estimated **G** matrices separately for each population. The full model in matrix notation was as follows:

(1) $$y_{\mathrm{ijkl}}=\mu +B_{i}+S_{j}+D_{k(j)}+e_{l(ijk)}$$

*μ* and *B*(*i*) are the fixed effects of intercept and block, *S_j_* represents sires, *D_k(j)_* represents dams within sires, and *e_l_*_(_ *_ijk_*_)_ represents the residual variance. For our design, *i* ranges from 1 to 3 for the blocks, *j* from 1 to 50 for sires, and *k* from 1 to 3 for dams within sires.

For some of the subsequent analyses, we removed the fitness proxy traits or treated them separately (noted explicitly below). We present results from analyses using traits standardized by the global standard deviation, following Hine et al. (2009) and Gosden and Chenoweth (2014). Conclusions from the standardized and unstandardized analyses did not differ in statistical or biological significance (McGoey, 2019).

### 2.8 Comparison of G matrices: overview

Researchers have used many different methods to compare **G** matrices (see Aguirre et al., 2014; Calsbeek & Goodnight, 2009; Roff et al., 2012). Because there are more than a dozen methods for **G** matrix comparison, with different strengths and weaknesses (see, e.g., Aguirre et al., 2014; Puentes et al., 2016), we used several methods with complementary strengths and weaknesses. We sought methods that were biologically interpretable and mathematically tractable, and where statistical uncertainty could be estimated. The biological implications of some metrics can be difficult to ascertain, so here we emphasize methods with clear links to the evolution of the populations (Aguirre et al., 2014).

To examine overall differences in matrix attributes (i.e., size, shape, orientation), we used Krzanowski's common subspace analysis and the fourth‐order genetic covariance tensor. Krzanowski's method determines whether the majority of genetic variance in trait space is common among populations. The subspace analysis allows an investigation whether the leading directions of multivariate trait space (i.e., trait combinations that explain the most variance in the data) are in common among a set of matrices. Put broadly, it characterizes whether the leading principal components are in common among matrices. As described by Blows et al. (2004), this approach is more complete than comparing the angles of the leading PCs, because it accounts for the possibility that PC1 of one matrix or population may in fact be very similar to PC2 of another. In contrast, the tensor analysis characterizes patterns of variation (or divergence) in matrices. It can identify the trait combinations with the greatest difference in genetic variance among populations, and, when integrated with a Bayesian framework, allow for an investigation of all the variation in **G** matrices among populations while taking into account uncertainty in **G** matrix estimates.

To examine the implications of **G** matrix divergence for the evolutionary trajectories of the populations, we solved the multivariate breeder's equation and used the R metric, which predicts evolution with and without covariances (Agrawal & Stinchcombe, 2009). Solving the breeder's equation allows an estimation of whether the observed **G** matrices will produce different responses to selection when confronted with a single, empirically estimated regime of selection. In contrast, the R metric evaluates whether the observed patterns of genetic covariances among traits will slow or accelerate the response to selection. We also used random skewers (Cheverud and Marroig, 2007), in which G matrices are multiplied by a large universe random selection vector (the skewer) and the vector correlation or the angle of the resulting response vectors is calculated; these results are presented and discussed in the Supporting Information. Collectively, the approaches we implement examine the shared geometry of **G** matrices (Krzanowski), directions in multivariate space by which they diverged (fourth‐order tensors), how they affect the response to selection (breeder's equation, skewers), and whether they constrain adaptation (R metric). These methods provide a thorough analysis of any divergences between **G** matrices, and can point to the traits underlying these differences, and the implications for future responses to selection. Specific details of each method are outlined below.

#### 2.8.1 Krzanowski's common subspace analysis

Some parts of multivariate trait space will have genetic variance, while others will not. We can examine whether the subspaces with the most genetic variation are similar for multiple populations using the Krzanowski subspace analysis (Krzanowski, 1979). In other words, are the directions in multivariate space which contain the most genetic variance the same for each population? To find the subspace of most similarity among *p* populations (*t* = 1, …, 6 (in our case)), we used the equation:

$$H=\sum_{t=1}^{p}A_{t}A_{t}^{T}$$

where matrix transposition is indicated by the superscript *T*, and the subset *k*_t_ of the eigenvectors of *G*_t_ are contained in *A*_t_ (Aguirre et al., 2014). The number of eigenvectors included in the summary matrix **H** is half the total number of traits that were examined (Aguirre et al., 2014; Puentes et al., 2016). Any eigenvalues of **H** that are less than *p* indicate that the directions of genetic variation described by that eigenvector differ among populations. In contrast, eigenvalues equal to *p* indicate common subspaces—that is, directions of genetic variation that can be described by the same eigenvectors (Aguirre et al., 2014). The advantage of the Krzanowski method is its clear bounded statistic, which ranges from zero (most divergent) to *p* (most similar) (Blows et al., 2004). Although this method is restricted to examining the subspaces of **G** with the most variation, they are the subspaces that will bias responses to selection and therefore are the most relevant to future adaptation (Aguirre et al., 2014). We conducted the Krzanowski subspace analysis both using all traits (six traits, three eigenvectors) and while excluding fitness traits (four traits, two eigenvectors). To test for significance, we compared results with those from the randomizations of phenotypes to sires and dams. To consider subspaces significantly diverged, an **H** value had to be lower than *p*, and it had to be lower than the 95% HPD interval calculated from the null (randomized) **G** matrices.

#### 2.8.2 Genetic covariance tensor

Much as principal components can be used to define directions in multivariate space that contain the most variation in traits, eigentensors can be used to describe differences among matrices, in this case, **G** matrices. The tensor method examines the differences between multiple matrices by using eigenanalyses (Aguirre et al., 2014; Hine et al., 2009; also see Fig. 2 of Walter et al., 2018). We briefly describe the execution of this analysis; interested readers can refer to Hine et al. (2009), Aguirre et al. (2014), and Walter et al. (2018) for more information on the advantages of this approach and details of its implementation. A tensor of the fourth order is required since matrices (second order) are being compared ($\sum_{\mathrm{ijkl}}=cov\left(G_{\mathrm{ij}},\;G_{\mathrm{kl}}\right))$. The tensor can be represented as the matrix **S**, containing variances and covariances of all the elements among the **G** matrices—that is, the variances and covariances of genetic variances and covariances (Aguirre et al., 2014; Hine et al., 2009). Analogous to decomposing a matrix into eigenvalues and eigenvectors, the fourth‐order tensor can be decomposed into eigenvalues and second‐order eigentensors, which indicate how the **G** matrices have diverged from one another (Aguirre et al., 2014; Hine et al., 2009). Eigentensors with larger eigenvalues describe differences in the genetic variances and covariances among populations. These eigentensors can themselves be subjected to eigenanalysis, to determine the trait combinations (eigenvectors) that lead to the differences captured by the eigentensors (Walter et al., 2018).

The tensor method has the advantages of being compatible with various experimental designs and encompassing all the variation among matrices (Aguirre et al., 2014). Unlike some other methods that may underscore differences that do not easily relate back to the original traits, the fourth‐order tensor allows for the identification of specific trait combinations that have different variances in the study populations (Aguirre et al., 2014). We used the tensor method on both standardized **G** matrices containing all six traits and just the four phenotypic traits, using code modified from Aguirre et al. (2014) and Puentes et al. (2016). To determine whether an eigentensor described significant variation among populations, we again took advantage of the Bayesian framework (Aguirre et al., 2014). To test whether variation among populations was larger than what could be expected by random sampling error, we compared the posterior distribution of each eigenvalue to the distributions generated from the randomized (null expectation) populations (Aguirre et al., 2014; Careau et al., 2015; Walter et al., 2018). We considered an eigentensor to encompass biologically meaningful variation among populations if the variance it explained was higher than the 95% HPD values calculated from the randomized **G** matrices (null expectations).

#### 2.8.3 Solving the breeder's equation

One of the primary motivations of estimating **G** matrices in the first place was to use them to predict responses to selection. The multivariate breeder's equation method uses selection estimates taken empirically to do just that. The trait responses of a population facing a realistic selection scenario can then be generated (Lynch & Walsh, 1998). Solving the breeder's equation with a single selection vector can be used to compare **G** matrices: Do the matrices lead to significant differences in response to an observed pattern of selection (Stinchcombe et al., 2009)? Significant differences between populations can be assessed by examining overlaps between the 95% HPD intervals of the predicted response to selection.

To implement this, we used two approaches. First, we used field data to obtain point estimates of the elements of *β* by performing a multiple regression of each fitness metric on the phenotypic traits using sire means. We used global sire means to obtain a common representation of the overall pattern of directional selection experienced by the field experimental population (estimating selection for each population separately would make it impossible to distinguish whether differences in observed responses were due to **G** or the selection estimates). We used the first principal component of seed mass and total inflorescence size as a composite fitness component; separate analyses of male and female fitness revealed similar results and are not presented. In these analyses, we held *β* fixed at point estimates from the multiple regression; differences in predicted responses to selection thus come from differences in the **G** matrices between each population. Each posterior sample of G in each population was multiplied by *β* to obtain a posterior distribution for Δ $\bar{z}$; we compare populations by examining differences in the means and 95% HPD intervals of Δ $\bar{z}$. To ensure that units were comparable, we performed calculations of unstandardized G matrices (units of traits^2^ for variances) and unstandardized selection gradients (units of traits^−1^) to obtain predicted responses to selection in original trait units. We then divided these responses to selection by the global standard deviation of each trait, to put each estimate of Δ $\bar{z}$ into comparable standard deviation units.

In the second approach, we incorporated uncertainty in our estimates of *β* by implementing a Bayesian regression approach with the bas.lm function in R. We explored two priors (ZS‐null and AIC), and found that it did not affect the results, and report results using the ZS‐null prior. We performed 10,000 MCMC iterations to sample the posterior of the elements of *β*. We then combined a single posterior estimate of **G** with a single posterior estimate of *β* to estimate the response to selection, incorporating uncertainty in both components parts by sampling each of them from their posterior distributions.

#### 2.8.4 R values: predicting evolution with and without covariances

Covariances between traits can have important impacts on evolutionary trajectories by either constraining or accelerating adaptation. If introduced populations are subject to greater constraints to adaptation because of genetic covariances, this should be seen in lower rates of adaptation due to covariances, compared with native populations. Agrawal and Stinchcombe (2009) developed a summary statistic to quantify the impact of covariances on the adaptive potential of populations. Given a level of variance, the R metric can evaluate the effect of correlations on the rate of adaptation. Evolutionary biologists have often emphasized constraint in the context of genetic correlations, but in their discussion of the R metric, Agrawal and Stinchcombe (2009) argue this may not be warranted. Investigating correlations in more datasets can elucidate the prevalence of constraints from genetic correlations.

To implement this method, we calculated the rate of adaptation given the observed **G** matrices and constructed **G** matrices where there are no correlations between traits (i.e., all off‐diagonals are changed to zero). The “R value” is the ratio of the two. When R > 1, adaptation is accelerated by covariances between traits, and when R < 1, adaptation is slowed down by genetic correlations.

As with the breeder's equation method, the R metric requires selection gradients. We ran separate analyses for male and female fitness, as well as a composite of the two, to calculate selection on the four nonfitness traits. To compare populations, we again took advantage of the Bayesian framework and the 10,000 estimates of each **G** matrix. We compared the R values to determine whether the HPD intervals overlapped for each pairwise comparison of populations.

## 3 RESULTS

### 3.1 Differences in means

Using a traditional MANOVA, we found that there were significant differences between continents in the six phenotypic traits (*F*_4,1531_ = 41.150, *p* < 0.0001) and populations nested within continents (*F*_6,6136_ = 55.41, *p* < 0.0001), indicating multivariate divergence in the traits (Figure S1). Based on this, we considered continents and populations separately in further analyses of heritability and **G** matrices.

### 3.2 Univariate comparisons

**FIGURE 2:**
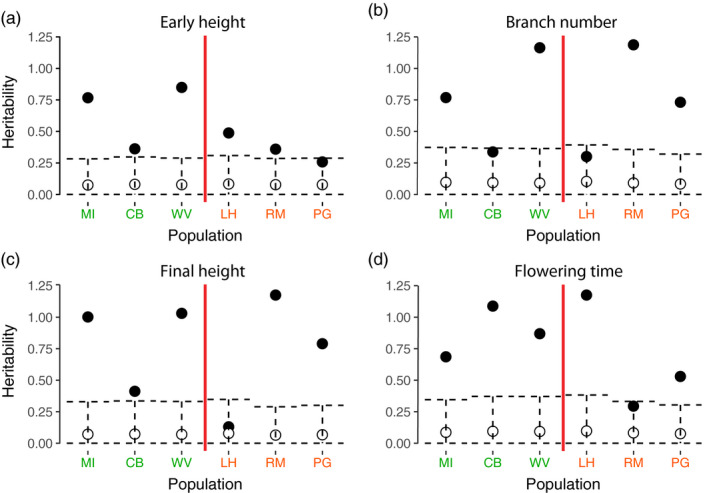
Heritability estimates of ragweed (a) early height, (b) branch number, (c) final height, and (d) flowering time. North American populations are to the left of the red line (from north to south: MI, CB, WV), and European populations are to the right of the red line (from north to south: LH, RM, PG). Mean posterior estimates are shown in black (circles), and randomized mean estimates are white circles with the 95% intervals shown as dashed lines. For heritability estimates, the genetic variance within each population is divided by the phenotypic variance for that population, rather than for the total experiment

Heritability estimates were higher than expected from sampling error for almost all traits and populations (see Figure 2). In some cases, estimates of heritability exceeded 1, which can happen due to sampling error (Hill & Thompson, 1978). While the variances associated with terms in the model sum to the phenotypic variance, *V*_A_ is 4x the sire variance, *V*_S_, and thus, narrow‐sense heritabilities can exceed 1 after *V*_S_ is multiplied by 4.

### 3.3 G matrix comparisons

#### 3.3.1 Krzanowski's common subspace analysis

**FIGURE 3:**
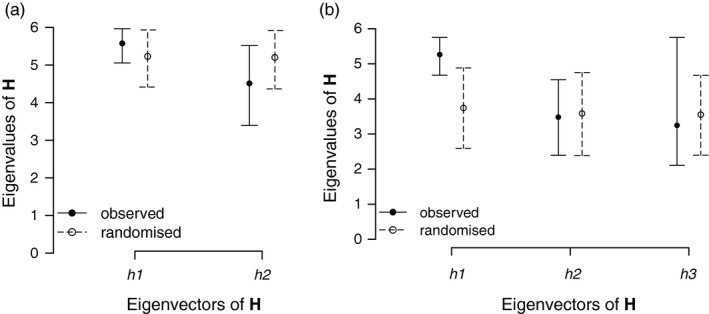
Results from the Krzanowski analysis using only the four phenotypic traits (early height, final height, branch number, and flowering time) (a) and including estimates for male and female fitness (b). The eigenvalues (mean and 95% HPD interval) of each of the first two (a) or three (b) eigenvectors of **H** are shown for the observed (closed circle, solid lines) and randomized (open circles, dashed lines). Values closer to 6 (the number of populations compared) indicate greater similarity in multivariate directions of genetic variation

We did not see significant divergences among subspaces among populations (Figure 3). This was true both when all six traits were included and when we excluded the fitness traits. In other words, the subspace containing the majority of genetic variation for the six populations was common: There is no evidence of population divergence in the multivariate space described by the leading principal components.

#### 3.3.2 Fourth‐order genetic covariance tensor

**FIGURE 4:**
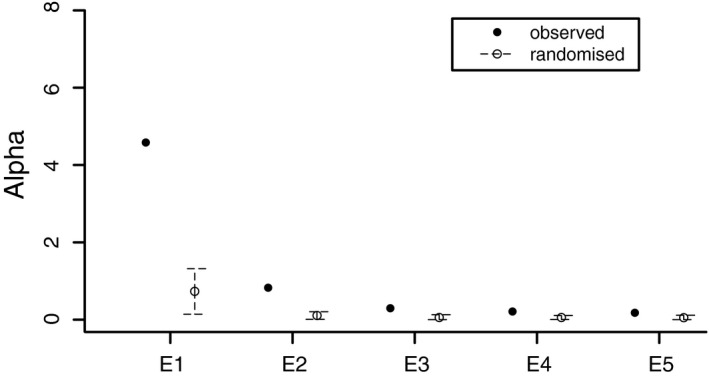
Results of tensor analysis of G matrices for six *A*.* artemisiifolia* populations. Eigenvalues of eigentensors for posterior mean **S** (the covariance matrix representing the fourth‐order covariance tensor). The amount of variance (alpha) accounted for by each eigentensor is shown for **G** matrices of the six observed (solid circle) and randomized (dashed line and open circle) populations. The error bars are the 95% HPD intervals generated using 500,000 MCMC total iterations for 1000 randomized phenotypes

For the tensor analysis using all six traits and six populations, we found two significant eigentensors (Figure 4)—that is, two directions describing variation and covariation among the six **G** matrices. The first eigentensor described the majority of variation (65%) among the **G**, but there was large uncertainty in this dimension. The West Virginian population was the most divergent in terms of each populations’ contribution to the first eigentensor (Figure S2). The first eigenvector of the first eigentensor (*e*11) accounted for 89% of the variation in this eigentensor, while the second and third eigenvectors explained 4% and 3.8% of the variation in the first eigentensor. The second eigentensor described 12% of the variation among **G** matrices. The first eigenvector of the second eigentensor accounted for 47.5% of the variation in this eigentensor. Altogether, the first three eigenvectors of the second eigentensor accounted for 82% of its variation.

#### 3.3.3 Solving the breeder's equation

**FIGURE 5:**
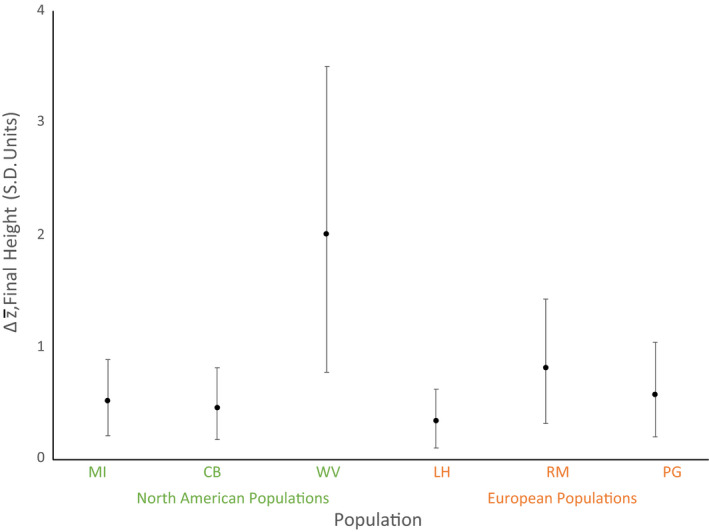
Predicted response to selection for final height predicted by solving the breeder's equation for six ragweed populations (three native (MI, CB, and WV) and three introduced (LH, RM, and PG)). Final height is shown in standard deviation units

Estimated selection gradients differed in magnitude but not direction depending on the fitness metric that we used (female fitness, male–female, or a composite of the two). Results from solving the breeder's equation were consistent across fitness metrics, so we only present those using composite fitness here (Figure 5). We examined the results of pairwise comparisons for six populations for responses in four nonfitness traits. Only final height showed a significant difference in pairwise comparisons. As with the random skewers methods (Supporting Information), there were not more divergent pairs between continents compared with within continents, indicating that this result is not due to the use of a single selection gradient rather than a large universe of them. In all cases where we saw a difference between populations, one of the populations involved was West Virginia. While these data do not suggest significant differences among populations in the likely response to selection, we did predict significant evolutionary responses in many cases. These data indicate that while we predict significant evolutionary responses (i.e., the strength of covariances do not make the predicted response to selection indistinguishable from zero), there is no heterogeneity among predictions based on populations or continent of origin.

#### 3.3.4 R values: predicting evolution with and without covariances

**FIGURE 6:**
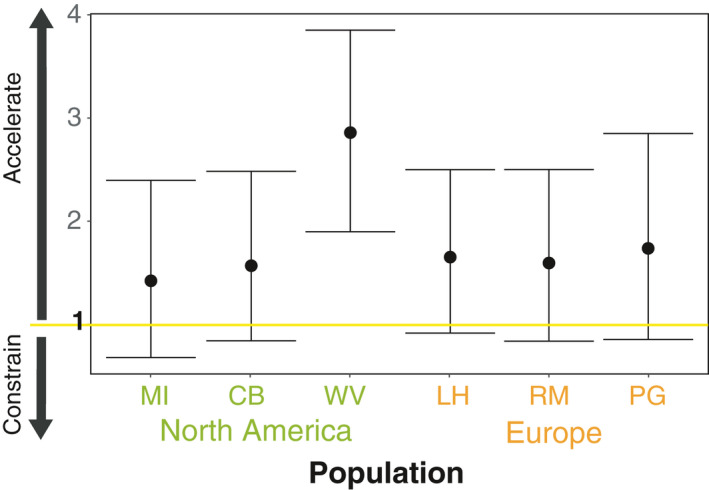
R metric for three native (MI, CB, and WV) and three introduced (LH, RM, and PG) ragweed populations. Values greater than 1 indicate that evolution would be accelerated by genetic correlations, and values less than 1 indicate they would be constrained by genetic correlations. Error bars depict the 5th and 95th percentile

We observed that the point estimates of the R metric were positive: In other words, the rate of adaptation is accelerated by genetic covariances, relative to not having them (Figure 6). Individual point estimates are moderate (~1.5, where one indicates no effect of covariances on adaptation), with the exception of the West Virginia population. In this case, the point estimate is close to 3, and with uncertainty that does not include 1, the rate of adaptation is threefold faster with covariances than compared to when they were absent. In general, this occurs when the direction of selection and the sign of genetic covariances are concordant: traits selected in the same direction when positively correlated, or in opposite directions when negatively correlated. As with the multivariate breeder's equation, we failed to see any consistent differences between the native and invasive range.

## 4 DISCUSSION

Invasive species are an important component of anthropogenic global change (Simberloff, 2014). Invasion genetics examines the importance of genetic factors in determining the trajectory that an invasion will take (Barrett, 2015). Most traits that will be important for a response to selection in new habitats will be quantitative (Dlugosch & Parker, 2008a; Estoup et al., 2016), which has led to numerous calls for invasion research from a quantitative genetics perspective (Bacigalupe, 2009; Lawson Handley et al., 2011) and direct comparisons of additive genetic variance between native and introduced populations (Barrett, 2015). We used a common garden experiment paired with multivariate Bayesian analyses of additive genetic (co)variation to compare the quantitative genetic architecture for native and introduced ragweed populations. While we found some differences in phenotypic traits and their genetic variances, the dominant picture that emerges is that **G** matrices of introduced populations were not significantly or homogeneously diverged from native populations of ragweed. We found that introduced populations did not have lower additive genetic variance or diminished adaptive capacity when compared to native populations. Below we discuss the implications of these results for understanding ragweed's invasion in particular, and more generally the stability of **G** through space and time.

### 4.1 Quantitative variation and ragweed invasion

Invasive species represent a major global economic and ecological concern (Pimentel et al., 2005). The field of invasion biology originally emerged from community ecology and emphasized ecological indicators over evolutionary aspects of introduced populations (Davis, 2010). Treating invasive species as static entities may lead to poor predictions on how invasions will proceed (Whitney & Gabler, 2008) since evolutionary change occurs on ecological timescales (Thompson, 1998). Understanding the role that evolutionary factors, such as genetic diversity, play in the invasion process is important to our ability to assess and contain invasions (Sakai et al., 2001).

Like many weedy plants, common ragweed has benefited immensely from anthropogenic changes to natural landscapes (Bassett & Crompton, 1975; Lavoie et al., 2007). Ragweed is thought to be native to the plains of North America but has spread across the globe (Bassett & Crompton, 1975). Humans are implicated in every step of this process, from physically transporting it across oceans as a grain contaminant, to constructing roads, to providing consistent disturbances, which allow ragweed (an otherwise poor competitor) to persist (Chauvel et al., 2006; Kiss & Béres, 2006; Lavoie et al., 2007; MacKay & Kotanen, 2008). Recent anthropogenic climate change has extended the growing season for ragweed (Ziska et al., 2011). The consequences of ragweed invasion in Europe are multipronged, including impacts on human health, agricultural productivity and ecological integrity (Buttenschøn et al., 2010; Chauvel et al., 2006). It has been highlighted as a weed of particular concern, with much effort devoted to research on its spread and eradication effort (Pinke et al., 2011).

Ragweed appears to have evolved rapidly in its introduced range, and our results suggest that it has sufficient quantitative genetic variation for adaptation in traits that could allow further expansion of its range and abundance in Europe. Clines in flowering time and reproductive biomass, and a high *Q*_ST_ (vs *F*_ST_) value for reproductive allocation suggest that ragweed has locally adapted across Europe (Chun et al., 2011; Hodgins & Rieseberg, 2011; McGoey et al., 2020; van Boohemen et al., 2018; van Boheemen & Hodgins, 2020). Our results illustrate that the combination of introduction, founder events, and recent adaptation has not reduced quantitative genetic variation relative to source populations: Introduced populations had neither lower heritabilities nor divergent **G** matrices, nor greater evolutionary constraint due to genetic covariances. Like many other studies, there was reasonable uncertainty around our **G** estimates, which is a potential qualifier on our conclusion that there is minimal divergence in the **G** matrices between populations (Puentes et al., 2016). Power and sample size challenges are inherent to the **G** matrix approach. In addition to large uncertainty due to estimation of sire variances (rather than characterizing individual‐level traits with much greater sample sizes), the multivariate framework introduces many more variances and covariances to be estimated, a challenge that gets harder with each additional trait considered (parameters estimated = $\frac{n\;\times \;n+1}{2}$ for *n* traits).

The quantitative genetic variation we found is especially concerning given that ragweed also has several characteristics recognized as advantageous for invasion. Ragweed has a short generation time, small propagule size, and high propagule pressure, all of which will facilitate its spread (Dormontt et al., 2010; Novak, 2007; Whitney & Gabler, 2008). Outcrossing introduced plants with substantial genetic variation has the capacity to rapidly adapt to their new circumstances (Colautti & Barrett, 2013). The traits we focused on size, timing, and architecture traits are likely critical to the ability of ragweed to continue its range expansion in Europe. Both native and invasive ranges appear to be restricted by phenology (Chapman et al., 2013). Genetic variation for size and flowering time is critical to the ability of invasive species to establish and spread (Colautti & Barrett, 2013). The spread of ragweed has been facilitated by railroads and highways, which act as both corridors and habitat (Kiss & Béres, 2006; Lavoie et al., 2007). Together, the interconnectedness of Europe and the high levels of genetic variation already on the continent could accelerate the spread of ragweed into new areas. Eradication efforts of ragweed populations must take into account the likelihood of adaptation in response to any interventions and should never treat invasive populations as static.

### 4.2 Divergences between G matrices and their implications

Evolutionary biologists have long held an interest in the stability of **G** over space and time, since the ability to predict evolutionary trajectories are contingent on **G** matrix consistency (Arnold et al., 2008). **G** matrices will be impacted by mutation, selection, drift, recombination and migration (Arnold et al., 2008). The complexities of all these forces interacting have meant that theoretical predictions for how **G** will change over time have been intractable and the dynamics of **G** must be studied empirically (Revell, 2007; Turelli, 1988). There have been several empirical and simulation studies on the stability of **G**, but results are equivocal, and the difficulty in rigorously estimating one **G** matrix, let alone multiple **G** matrices, has meant that we do not yet have a clear picture of how **G** varies in space and time (Aguirre et al., 2014; Arnold et al., 2008; Delahaie et al., 2017). The advent of statistical methods that allow for rigorous comparison of multiple **G** matrices—while accounting for uncertainty in each—has increased the impetus and utility of more empirical research on **G** matrix variability (Delahaie et al., 2017). Despite their importance, studies of **G** matrix variation remain rare, especially for nonmodel organisms (Cano et al., 2004; Delahaie et al., 2017) and spatial variability is even less well explored than changes through time (Puentes et al., 2016).

Introduced populations could face two main forces that could shift **G** when compared to native populations. First, a bottleneck could cause a shift in the genetic architecture (Whitlock et al., 2002). Second, the populations could face strong selection, which could alter **G** (Arnold et al., 2008). The invasion of ragweed into France has been characterized by multiple introductions and admixture (Genton et al., 2005; van Boohemen et al., 2017). Molecular markers show an equivalent or greater diversity in the introduced range, when compared to the native range (Genton et al., 2005; Li et al., 2012; McGoey et al., 2020). However, the absence of a bottleneck detected from neutral makers does not mean there could not be shifts in quantitative genetic architecture: Neutral markers are not useful as proxies for quantitative genetic variation (Mittell et al., 2015; Reed & Frankham, 2001). For example, Eroukhmanoff and Svensson (2011) investigated differences in the **G** matrices of two ecotypes of aquatic isopods. In two different lakes, the isopods have colonized a new habitat in the last few decades. While Eroukmanoff and Svensson (2011) found no difference in neutral genetic variation, additive genetic variance decreased by nearly 50% (Eroukhmanoff & Svensson, 2011). Likewise, we cannot use neutral markers to assess adaptive potential. In their study of *Hypericum canariense*, Dlugosch and Parker (2008b) found rapid adaptation of important life history traits in invasive populations, despite large bottlenecks and low molecular genetic diversity.

Our findings, along with past studies (Hodgins & Rieseberg, 2011), reveal genetic differentiation for mean values of quantitative traits in ragweed's introduced range, consistent with divergent directional selection since colonization. There have also been multiple introductions from different source populations, with the potential to cause shifts in **G** due to waves of migration. Ragweed's habitat preference for recently disturbed sites suggests that the species often experiences frequent bottlenecks in both its native and introduced ranges, which could introduce periodic bottlenecks to all populations. The effects of regular bottlenecks might be ameliorated by its prodigious pollen production, wind pollination with wide pollen dispersal, and outcrossing mating system. Despite the different evolutionary forces introduced ragweed populations have faced, all of which could have contrasting effects on levels of quantitative genetic variation, their **G** matrices have not substantially diverged from those of native populations. It may be that the interplay between these forces and the genetic architectures of complex traits such as size and phenology leads to a relatively stable **G**; alternatively, it may be that species such as ragweed become invasive because they have a relatively stable **G** that can lead to responses to selection new ranges despite the interplay of these forces.

We used a variety of methods to assess the magnitude of **G** matrix differences for native and introduced ragweed populations. Overall, the **G** matrices are largely stable across geography, consistent with studies on other taxa that have also found similarity in **G** across conspecific populations. While we did find some moderate differences between **G** matrices, most differences seem to be driven by the West Virginian population, which was highlighted by several of the analyses as a divergent population. Differences were not more apparent between populations from different continents than those from the same range. When confronted with the same selection scenario, responses of introduced populations would not be more different from native populations than from each other. There are too few studies of **G** matrix variability among populations for broad patterns to emerge, but past authors have argued that **G** matrices are stable across geography (Arnold et al., 2008; Delahaie et al., 2017), and our results are consistent with that interpretation.

## 5 CONCLUSION

It is increasingly appreciated that evolutionary factors are important in the invasion process and that there is value in approaching the study of invasive species from a quantitative genetics perspective. Data on the adaptive potential of wild populations are scarce (Delahaie et al., 2017), but are necessary to understanding evolution in natural environments.

We used a multivariate Bayesian approach and found that introduced *A*.* artemisiifolia* populations are not limited in their adaptive potential when compared to native populations. Importantly, the availability of additive genetic variance seen here indicates that ragweed will be able to respond to selection pressures in the introduced range, whether from novel selection, global change, or eradication efforts. Combined with its annual life history and prolific production of seeds, ragweed is primed to adapt rapidly to selection pressures that arise in its introduced range.

## CONFLICT OF INTEREST

The authors declare no conflict of interest.

## Supporting information

Supplementary Material

## Data Availability

Data for this study have been deposited in the DRYAD repository: https://doi.org/10.5061/dryad.sj3tx964d.
