# Supplementary material for: Introduced populations of ragweed show as much evolutionary potential as native populations
Source: Evol Appl. 2021 Apr 2;14(5):1436–49. doi: 10.1111/eva.13211 (PMC8127702; doi:10.1111/eva.13211)
Supplement: Supplementary file 1 — Supplementary Material [file EVA-14-1436-s001.docx]

**Supplemental Material**

The table of contents is created in word. On a PC, use control-click on the text below to be brought directly to that item in the document.

Contents

[Figure S1: Phenotypic distributions of traits by population and continent. 2](#_Toc55820455)

[Figure S2: Coordinates of the posterior mean of the G matrices in the space of the first two Eigentensors. 3](#_Toc55820456)

[Table S1: Trait combinations for E1 and E2 for the tensor analysis of six traits and six ragweed populations 4](#_Toc55820457)

[Results of random skewers analysis of the G matrices 5](#_Toc55820458)

[Table S2: Summary statistics for the estimated univariate traits 8](#_Toc55820459)

[Table S3a: Estimates G matrices 10](#_Toc55820460)

[Table S3b: Posterior 95% HPD intervals for the G matrices. 13](#_Toc55820461)

***
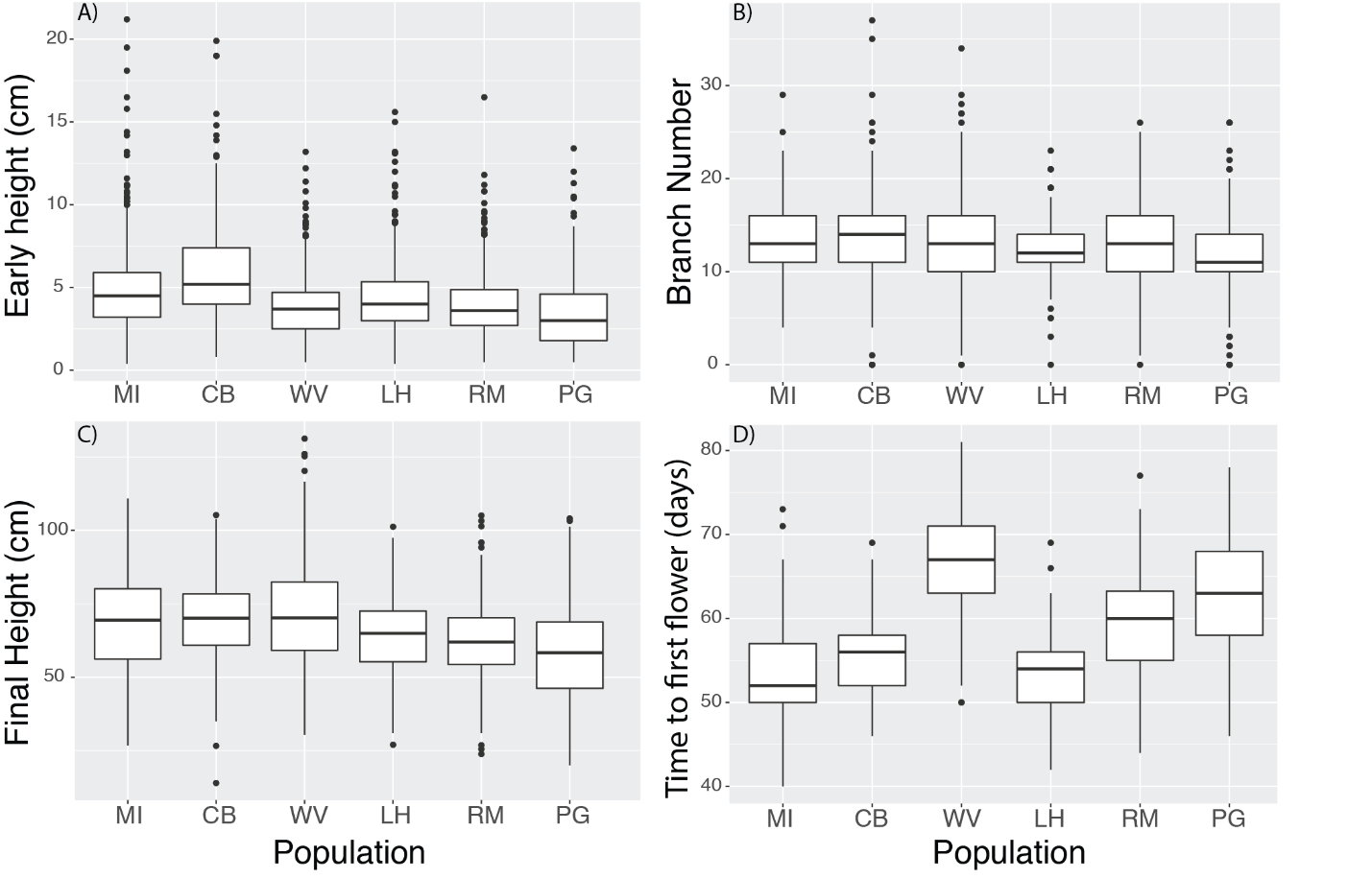
***

# Figure S1: Phenotypic distributions of traits by population and continent.

Phenotypic traits of six populations (three native populations from north to south followed by three introduced populations form north to south). Early height (A), branch number (B), final height (C) and flowering time (D).


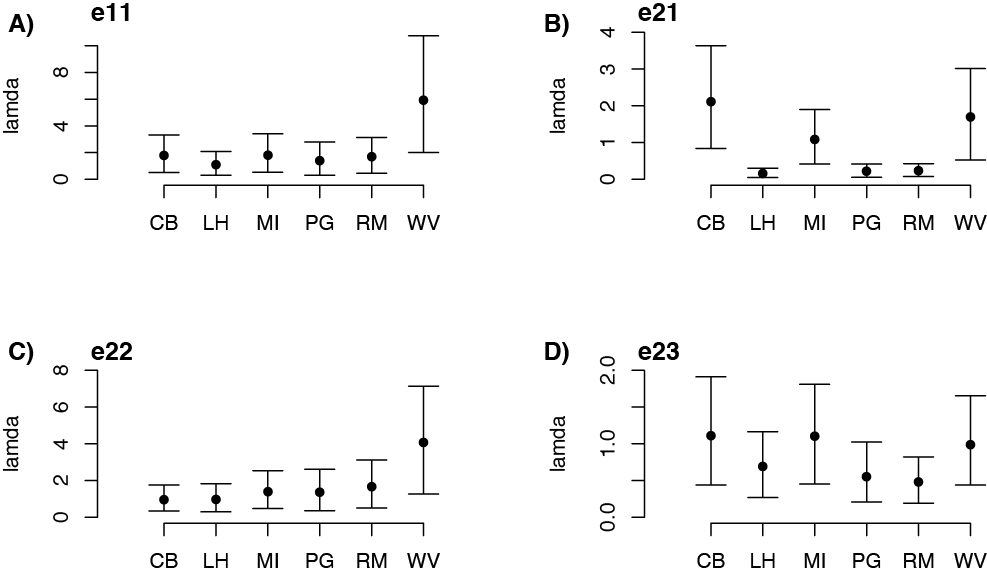


# Figure S2: Coordinates of the posterior mean of the G matrices in the space of the first two Eigentensors.

Coordinates of posterior mean for six standardized *A. artemisiifolia* **G** matrices in the space of the first eigentensor (E1) and second (E2) eigentensors. The first eigentensor (E1) described the majority of variation (65%) among the **G** matrices. The first eigenvector of the first eigentensor (*e*11) accounted for 89% of the variation in this eigentensor. The second eigentensor (E2) described 12% of the variation among **G** matrices. The first eigenvector of the second eigentensor (*e*21) accounted for 47% of the variation in this eigentensor. Altogether, the first three eigenvectors of the second eigentensor (*e*21, *e*22, *e*23) accounted for 82% of its variation. Error bars are the 95% HPD intervals for 10000 MCMC sample

# Table S1: Trait combinations for E1 and E2 for the tensor analysis of six traits and six ragweed populations

Trait combinations for **E**1 and **E**2 for the tensor analysis of six traits and six ragweed populations

|  | *S.eigval* | eT.val | Flowering  time | Final  height | Branch  number | Early  height | Female  fitness | Male  fitness |
| --- | --- | --- | --- | --- | --- | --- | --- | --- |
| e1.1 | 4.584 | -0.998 | 0.072 | -0.527 | -0.517 | -0.199 | -0.408 | -0.492 |
| e1.2 | 4.584 | -0.046 | 0.341 | 0.374 | 0.500 | 0.059 | -0.368 | -0.596 |
| e1.3 | 4.584 | 0.043 | 0.260 | 0.079 | 0.100 | -0.937 | 0.178 | 0.079 |
| e1.4 | 4.584 | -0.024 | -0.573 | 0.518 | -0.215 | -0.230 | -0.537 | 0.126 |
| e1.5 | 4.584 | -0.007 | -0.538 | -0.515 | 0.638 | -0.151 | -0.122 | -0.036 |
| e1.6 | 4.584 | -0.001 | 0.440 | -0.206 | 0.138 | 0.057 | -0.602 | 0.616 |
| e2.1 | 0.824 | -0.871 | 0.056 | -0.010 | -0.024 | -0.194 | -0.978 | -0.052 |
| e2.2 | 0.824 | 0.367 | 0.266 | -0.690 | -0.626 | 0.066 | 0.037 | -0.236 |
| e2.3 | 0.824 | -0.264 | -0.025 | -0.172 | 0.021 | 0.757 | -0.182 | 0.603 |
| e2.4 | 0.824 | -0.125 | 0.356 | -0.397 | 0.321 | -0.524 | 0.090 | 0.575 |
| e2.5 | 0.824 | 0.115 | 0.843 | 0.491 | -0.179 | 0.127 | 0.020 | 0.028 |
| e2.6 | 0.824 | -0.088 | 0.298 | -0.311 | 0.687 | 0.309 | -0.031 | -0.497 |

# Results of random skewers analysis of the G matrices

***Random skewers***

A primary motivation for comparing **G** matrices is to determine whether the evolutionary trajectories of populations will diverge, and the random skewers approach allows for an investigation of this question (Roff et al. 2012). We can ascertain the collinearity of the responses of two matrices to a series of randomly generated selection vectors (Cheverud and Marroig 2007). Since we had six populations, we did a series of pairwise comparisons (*i.e.,* each population was compared to each other population). We used two approaches (described below) to test for differences in the responses of populations to random skewers.

In the main text, we standardized each trait by its global standard deviation. As a consequence, the total amount of variation in each trait will be the same, although the relative amount of sire, dam, and environmental variance for each trait can of course vary from trait to trait. As a complement to those analyses, for our first set of analyses with random skewers we did not standardize traits, and as a consequence, traits can differ in both their total phenotypic variance (Vp) as well as the individual variance components (Vs, Vd, Ve). In a second set of analyses, we standardized the data as in the main text.

***Cheverud’s Approach: Angle between response vectors***

For each posterior sample of a **G** matrix, we generated 1000 random vectors, and calculated the multivariate response to selection for each population. For every pair of populations, we then calculated the vector correlation of the responses to selection, and the mean angle between these response vectors. We repeated this for every **G** matrix estimate in the posterior sample (i.e., treating each posterior sample as if it were the only estimate of **G**).

***Aguirre’s Modification: Differences in available genetic variance***

Aguirre and colleagues developed a method based on random skewers that compares the magnitude of genetic variances of multiple populations (Aguirre et al. 2014). In this approach, every randomly generated selection vector is projected through all the iterations of each **G** matrix to estimate genetic variance in the direction of the skewer (cf. Lin and Allaire 1977). For each vector, populations are considered to differ if their HPD-intervals do not overlap—i.e., they differ in their genetic variance in the direction of the skewer.

As with the first method, 1000 random selection vectors were generated. Each random selection vector was projected through every MCMC iteration of every **G** matrix to estimate genetic variance in the direction of the skewer (Lin and Allaire 1977; Aguirre et al. 2014); consequently for each of the 1000 random vectors, we had posterior distributions of the genetic variance. For each pair of populations, we determined if the highest posterior densities overlapped and collated the cases where they did not.

***Results***


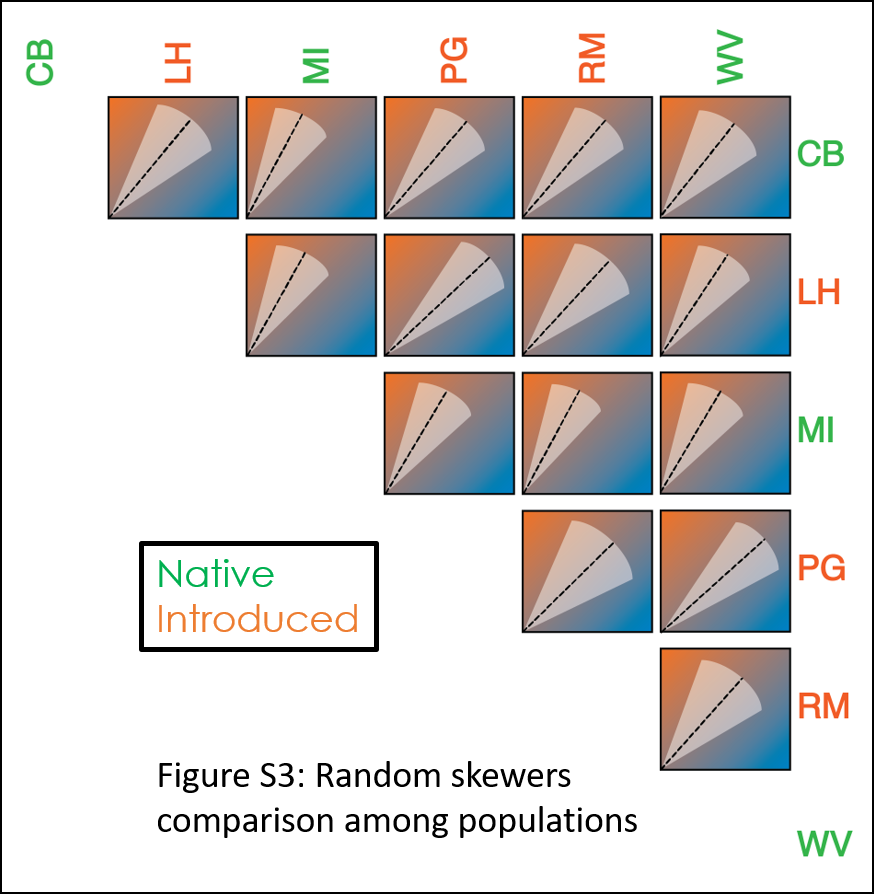
Unstandardized data. We saw mean angles of intermediate value for our one-by-one population comparisons (θ ranging from 0.45 to 0.65). For the Aguirre et al. method, we found that when there was divergence between two populations, one of the populations was almost always West Virginia (557 of 563 vectors). Our random skewers results consistently show that divergences between continents were not larger than for populations within continents (Figure S3).

Standardized data. We saw mean angles of intermediate value for pairwise population comparisons ranging from 0.16 to 0.33 degrees. For the Aguirre et al. (2014) method, we found that when there was divergence between two populations, one was always West Virginia. The random skewers results consistently show that divergences between the native and introduced ranges were not larger than for populations within each continent. The reduced angles for skewers applied to standardized data, compared to unstandardized data, suggests that some of the divergence described above is driven by differences in the total variance in traits differing between populations.

# Table S2: Summary statistics for the estimated univariate traits

Trait means, standard errors, sire, dam and error variance (σ2) components, heritabilities (h2, and 95% HPD) and sample sizes (number of half- and full-sib families, and the number of plants) for each of four traits (early height, final height, branch number, flowering time) for six populations (Native: MI, CB, WV, Introduced: LH, RM, PG).

| Population | Trait | Mean | Standard  Error | Sire σ | Δ DIC Sire | Dam σ | Δ DIC Dam | Error σ | *h^2^* | HPD  *h^2^* | No. Sires | No. Dams | No. Plants |
| --- | --- | --- | --- | --- | --- | --- | --- | --- | --- | --- | --- | --- | --- |
| MI | Early height | 5.04 | 0.16 | 1.68 | -4.311 | 2.328 | -48.546 | 4.745 | 0.768 | 0.146,  1.293 | 48 | 139 | 337 |
| CB | Early height | 6 | 0.17 | 0.788 | -0.908 | 1.266 | -8.992 | 6.641 | 0.363 | 0,  0.784 | 50 | 139 | 318 |
| WV | Early height | 3.79 | 0.1 | 0.829 | -5.76 | 0.737 | -24.764 | 2.331 | 0.85 | 0.256,  1.405 | 50 | 148 | 348 |
| LH | Early height | 4.42 | 0.13 | 0.654 | -2.412 | 0.968 | -17.997 | 3.742 | 0.488 | 0,  0.924 | 49 | 141 | 332 |
| RM | Early height | 3.95 | 0.11 | 0.406 | -0.139 | 1.718 | -78.14 | 2.39 | 0.36 | 0,  0.824 | 48 | 140 | 350 |
| PG | Early height | 3.39 | 0.11 | 0.289 | 0.554 | 2.93 | -238.354 | 1.265 | 0.258 | 0,  0.734 | 48 | 139 | 351 |
| MI | Final height | 68.65 | 1.06 | 69.427 | -1.912 | 101.255 | -68.295 | 106.401 | 1.002 | 0.144,  1.718 | 42 | 110 | 249 |
| CB | Final height | 69.72 | 0.97 | 23.392 | -0.672 | 59.659 | -21.813 | 143.835 | 0.412 | 0,  1.016 | 42 | 103 | 238 |
| WV | Final height | 71.29 | 1.21 | 93.871 | -1.142 | 186.165 | -154.692 | 83.995 | 1.031 | 0,  1.752 | 39 | 108 | 243 |
| LH | Final height | 64.35 | 0.83 | 5.217 | 0.395 | 83.455 | -83.733 | 71.206 | 0.131 | 0,  0.545 | 37 | 100 | 231 |
| RM | Final height | 62.82 | 0.89 | 64.454 | -1.039 | 78.673 | -80.125 | 76.168 | 1.176 | 0.369,  1.968 | 38 | 106 | 266 |
| PG | Final height | 58.18 | 0.89 | 44.131 | -1.451 | 94.69 | -96.363 | 84.993 | 0.789 | 0,  1.424 | 41 | 112 | 281 |
| MI | Branch No. | 13.22 | 0.26 | 3.251 | 0.974 | 5.544 | -42.714 | 8.114 | 0.769 | 0,  1.389 | 42 | 110 | 249 |
| CB | Branch No. | 13.86 | 0.32 | 2.062 | -1.359 | 2.957 | -4.59 | 19.468 | 0.337 | 0,  0.852 | 42 | 103 | 238 |
| WV | Branch No. | 13.41 | 0.33 | 8.145 | -1.125 | 9.291 | -57.287 | 10.553 | 1.164 | 0.314,  1.94 | 39 | 108 | 243 |
| LH | Branch No. | 12.84 | 0.2 | 0.691 | -0.533 | 3.205 | -39.933 | 5.299 | 0.301 | 0,  0.862 | 37 | 100 | 231 |
| RM | Branch No. | 13.03 | 0.26 | 5.562 | -4.809 | 4.219 | -34.922 | 8.97 | 1.187 | 0.466,  1.88 | 38 | 106 | 266 |
| PG | Branch No. | 11.8 | 0.25 | 3.146 | 0.451 | 8.982 | -145.163 | 5.063 | 0.732 | 0,  1.408 | 41 | 112 | 281 |
| MI | Days to flower | 53.49 | 0.36 | 5.493 | 0.07 | 14.808 | -88.631 | 11.785 | 0.685 | 0,  1.34 | 42 | 110 | 249 |
| CB | Days to flower | 55.52 | 0.32 | 6.672 | -3.529 | 6.775 | -38.499 | 11.081 | 1.088 | 0.281,  1.906 | 42 | 103 | 238 |
| WV | Days to flower | 67.08 | 0.37 | 7.593 | -1.527 | 18.999 | -155.063 | 8.363 | 0.869 | 0,  1.644 | 37 | 103 | 236 |
| LH | Days to flower | 53.5 | 0.3 | 6.195 | -4.091 | 4.498 | -26.156 | 10.393 | 1.175 | 0.439,  1.848 | 37 | 100 | 231 |
| RM | Days to flower | 59.93 | 0.4 | 3.198 | -0.013 | 30.141 | -228.104 | 10.132 | 0.294 | 0,  0.9 | 38 | 106 | 266 |
| PG | Days to flower | 62.94 | 0.39 | 5.786 | -0.322 | 17.472 | -64.211 | 20.487 | 0.529 | 0,  1.179 | 41 | 112 | 281 |

# Table S3a: Estimates G matrices

Posterior mean G matrices for three native (MI, CB, WV) and three introduced (LH, RM, PG) populations of ragweed. Note that individual traits were standardized to their global standard deviations prior to analysis.

| **MI** |  |  |  |  |  |  |
| --- | --- | --- | --- | --- | --- | --- |
|  | **Flowering time** | **Final height** | **Branch number** | **Early height** | **Female fitness** | **Male fitness** |
| **Flowering time** | 0.53953498 | -0.21202925 | -0.1664389 | -0.0933694 | -0.02601353 | -0.1139425 |
| **Final height** | -0.21202925 | 1.09462775 | 0.213 | 0.02089476 | 0.02489843 | 0.1101473 |
| **Branch number** | -0.16643892 | 0.21299998 | 0.8399145 | 0.12149214 | 0.34276382 | 0.4194273 |
| **Early height** | -0.0933694 | 0.02089476 | 0.1214921 | 1.23664071 | 0.19209177 | 0.1842099 |
| **Female fitness** | -0.02601353 | 0.02489843 | 0.3427638 | 0.19209177 | 0.9346199 | 0.4115212 |
| **Male fitness** | -0.11394253 | 0.11014733 | 0.4194273 | 0.18420985 | 0.41152121 | 0.8790068 |
|  |  |  |  |  |  |  |
| **CB** |  |  |  |  |  |  |
|  | **Flowering time** | **Final height** | **Branch number** | **Early height** | **Female fitness** | **Male fitness** |
| **Flowering time** | 0.53563772 | -0.03803565 | 0.07499254 | -0.1976363 | -0.19640742 | -0.10925123 |
| **Final height** | -0.03803565 | 0.65064654 | 0.13935307 | 0.2178494 | 0.23240834 | 0.18440703 |
| **Branch number** | 0.07499254 | 0.13935307 | 0.90219341 | 0.1967781 | 0.16270363 | 0.32978906 |
| **Early height** | -0.19763632 | 0.21784942 | 0.19677806 | 0.9255056 | 0.25091474 | 0.30887674 |
| **Female fitness** | -0.19640742 | 0.23240834 | 0.16270363 | 0.2509147 | 2.0107245 | 0.08222899 |
| **Male fitness** | -0.10925123 | 0.18440703 | 0.32978906 | 0.3088767 | 0.08222899 | 0.99146414 |
|  |  |  |  |  |  |  |
| **WV** |  |  |  |  |  |  |
|  | **Flowering time** | **Final height** | **Branch number** | **Early height** | **Female fitness** | **Male fitness** |
| **Flowering time** | 0.69350529 | -0.1581298 | -0.1788843 | -0.04901676 | -0.143655 | -0.2893689 |
| **Final height** | -0.15812983 | 2.1770246 | 1.5138182 | 0.59468393 | 1.0286555 | 1.3329689 |
| **Branch number** | -0.17888434 | 1.5138182 | 2.0933963 | 0.6170026 | 1.059498 | 1.384938 |
| **Early height** | -0.04901676 | 0.5946839 | 0.6170026 | 0.65032631 | 0.4966472 | 0.5751737 |
| **Female fitness** | -0.14365499 | 1.0286555 | 1.059498 | 0.49664716 | 1.3080874 | 1.1012066 |
| **Male fitness** | -0.28936887 | 1.3329689 | 1.384938 | 0.57517366 | 1.1012066 | 1.8818047 |
| **LH** |  |  |  |  |  |  |
|  | **Flowering time** | **Final height** | **Branch number** | **Early height** | **Female fitness** | **Male fitness** |
| **Flowering time** | 0.614889906 | -0.004009211 | -0.17192914 | -0.13887039 | 0.04427425 | -0.13663525 |
| **Final height** | -0.004009211 | 0.5473658 | 0.21678909 | 0.0236316 | 0.08589629 | 0.19073134 |
| **Branch number** | -0.171929143 | 0.216789091 | 0.62489973 | 0.15927177 | 0.07025238 | 0.31565624 |
| **Early height** | -0.138870392 | 0.023631601 | 0.15927177 | 0.64669876 | 0.02971097 | 0.09898202 |
| **Female fitness** | 0.044274254 | 0.085896293 | 0.07025238 | 0.02971097 | 0.1099733 | 0.08446121 |
| **Male fitness** | -0.13663525 | 0.190731342 | 0.31565624 | 0.09898202 | 0.08446121 | 0.70819679 |
|  |  |  |  |  |  |  |
| **RM** |  |  |  |  |  |  |
|  | **Flowering time** | **Final height** | **Branch number** | **Early height** | **Female fitness** | **Male fitness** |
| **Flowering time** | 0.737697694 | 0.12607979 | -0.18583235 | -0.005672223 | -0.10109283 | -0.1170058 |
| **Final height** | 0.126079792 | 1.08474829 | 0.49643373 | 0.228438696 | 0.09976573 | 0.3034681 |
| **Branch number** | -0.185832351 | 0.49643373 | 1.11624788 | 0.02835341 | 0.17354964 | 0.413933 |
| **Early height** | -0.005672223 | 0.2284387 | 0.02835341 | 0.544256996 | 0.03966623 | 0.0847645 |
| **Female fitness** | -0.101092831 | 0.09976573 | 0.17354964 | 0.039666232 | 0.16425665 | 0.1252092 |
| **Male fitness** | -0.11700583 | 0.3034681 | 0.413933 | 0.084764502 | 0.12520922 | 0.5453972 |
|  |  |  |  |  |  |  |
| **PG** |  |  |  |  |  |  |
|  | **Flowering time** | **Final height** | **Branch number** | **Early height** | **Female fitness** | **Male fitness** |
| **Flowering time** | 0.7232397 | -0.2183076 | -0.184013 | -0.1508781 | -0.1417358 | -0.1837257 |
| **Final height** | -0.2183076 | 0.8857531 | 0.2703253 | 0.1588964 | 0.1289357 | 0.2317237 |
| **Branch number** | -0.184013 | 0.2703253 | 0.8671731 | 0.1639882 | 0.1069258 | 0.3369935 |
| **Early height** | -0.1508781 | 0.1588964 | 0.1639882 | 0.5217849 | 0.0448295 | 0.1441077 |
| **Female fitness** | -0.1417358 | 0.1289357 | 0.1069258 | 0.0448295 | 0.1358996 | 0.1272368 |
| **Male fitness** | -0.1837257 | 0.2317237 | 0.3369935 | 0.1441077 | 0.1272368 | 0.5359953 |

# Table S3b: Posterior 95% HPD intervals for the G matrices.

HPD intervals for G matrices for six ragweed traits for three native (MI, CB, WV) and three introduced (LH, RM, PG) populations.

| **MI** |  |  |  |  |  |  |
| --- | --- | --- | --- | --- | --- | --- |
|  | **Flowering time** | **Final height** | **Branch number** | **Early height** | **Female fitness** | **Male fitness** |
| **Flowering time** | 0.205,0.949 | -0.645,0.168 | -0.555,0.182 | -0.5,0.308 | -0.422,0.342 | -0.496,0.245 |
| **Final height** | -0.645,0.168 | 0.467,1.877 | -0.275,0.733 | -0.53,0.581 | -0.49,0.556 | -0.379,0.642 |
| **Branch number** | -0.555,0.182 | -0.275,0.733 | 0.351,1.488 | -0.367,0.632 | -0.102,0.856 | -0.044,0.936 |
| **Early height** | -0.5,0.308 | -0.53,0.581 | -0.367,0.632 | 0.556,2.032 | -0.321,0.741 | -0.289,0.745 |
| **Female fitness** | -0.422,0.342 | -0.49,0.556 | -0.102,0.856 | -0.321,0.741 | 0.375,1.613 | -0.049,0.975 |
| **Male fitness** | -0.496,0.245 | -0.379,0.642 | -0.044,0.936 | -0.289,0.745 | -0.049,0.975 | 0.337,1.524 |
|  |  |  |  |  |  |  |
| **CB** |  |  |  |  |  |  |
|  | **Flowering time** | **Final height** | **Branch number** | **Early height** | **Female fitness** | **Male fitness** |
| **Flowering time** | 0.214,0.9 | -0.35,0.255 | -0.291,0.43 | -0.594,0.122 | -0.782,0.303 | -0.492,0.266 |
| **Final height** | -0.35,0.255 | 0.271,1.132 | -0.24,0.57 | -0.157,0.648 | -0.323,0.868 | -0.223,0.625 |
| **Branch number** | -0.291,0.43 | -0.24,0.57 | 0.354,1.556 | -0.253,0.689 | -0.512,0.893 | -0.156,0.877 |
| **Early height** | -0.594,0.122 | -0.157,0.648 | -0.253,0.689 | 0.375,1.541 | -0.411,0.989 | -0.156,0.846 |
| **Female fitness** | -0.782,0.303 | -0.323,0.868 | -0.512,0.893 | -0.411,0.989 | 0.854,3.455 | -0.648,0.816 |
| **Male fitness** | -0.492,0.266 | -0.223,0.625 | -0.156,0.877 | -0.156,0.846 | -0.648,0.816 | 0.399,1.709 |
|  |  |  |  |  |  |  |
| **WV** |  |  |  |  |  |  |
|  | **Flowering time** | **Final height** | **Branch number** | **Early height** | **Female fitness** | **Male fitness** |
| **Flowering time** | 0.228,1.216 | -0.89,0.481 | -0.899,0.434 | -0.417,0.289 | -0.743,0.403 | -0.968,0.336 |
| **Final height** | -0.89,0.481 | 0.886,3.764 | 0.336,2.854 | 0.029,1.231 | 0.062,2.132 | 0.195,2.585 |
| **Branch number** | -0.899,0.434 | 0.336,2.854 | 0.885,3.662 | 0.042,1.242 | 0.111,2.15 | 0.25,2.686 |
| **Early height** | -0.417,0.289 | 0.029,1.231 | 0.042,1.242 | 0.281,1.07 | 0.042,1.026 | 0.039,1.181 |
| **Female fitness** | -0.743,0.403 | 0.062,2.132 | 0.111,2.15 | 0.042,1.026 | 0.461,2.372 | 0.201,2.209 |
| **Male fitness** | -0.968,0.336 | 0.195,2.585 | 0.25,2.686 | 0.039,1.181 | 0.201,2.209 | 0.714,3.274 |
| **LH** |  |  |  |  |  |  |
|  | **Flowering time** | **Final height** | **Branch number** | **Early height** | **Female fitness** | **Male fitness** |
| **Flowering time** | 0.255,1.052 | -0.32,0.312 | -0.531,0.137 | -0.462,0.17 | -0.094,0.201 | -0.476,0.201 |
| **Final height** | -0.32,0.312 | 0.196,0.978 | -0.109,0.618 | -0.293,0.331 | -0.053,0.252 | -0.133,0.589 |
| **Branch number** | -0.531,0.137 | -0.109,0.618 | 0.214,1.138 | -0.152,0.511 | -0.076,0.256 | -0.058,0.74 |
| **Early height** | -0.462,0.17 | -0.293,0.331 | -0.152,0.511 | 0.285,1.077 | -0.104,0.175 | -0.228,0.448 |
| **Female fitness** | -0.094,0.201 | -0.053,0.252 | -0.076,0.256 | -0.104,0.175 | 0.035,0.206 | -0.068,0.266 |
| **Male fitness** | -0.476,0.201 | -0.133,0.589 | -0.058,0.74 | -0.228,0.448 | -0.068,0.266 | 0.283,1.23 |
|  |  |  |  |  |  |  |
| **RM** |  |  |  |  |  |  |
|  | **Flowering time** | **Final height** | **Branch number** | **Early height** | **Female fitness** | **Male fitness** |
| **Flowering time** | 0.264,1.299 | -0.375,0.604 | -0.721,0.295 | -0.338,0.309 | -0.309,0.079 | -0.494,0.211 |
| **Final height** | -0.375,0.604 | 0.397,1.85 | -0.069,1.197 | -0.114,0.66 | -0.111,0.354 | -0.093,0.772 |
| **Branch number** | -0.721,0.295 | -0.069,1.197 | 0.388,1.926 | -0.362,0.419 | -0.054,0.443 | -0.006,0.919 |
| **Early height** | -0.338,0.309 | -0.114,0.66 | -0.362,0.419 | 0.233,0.918 | -0.104,0.205 | -0.184,0.369 |
| **Female fitness** | -0.309,0.079 | -0.111,0.354 | -0.054,0.443 | -0.104,0.205 | 0.063,0.291 | -0.05,0.296 |
| **Male fitness** | -0.494,0.211 | -0.093,0.772 | -0.006,0.919 | -0.184,0.369 | -0.05,0.296 | 0.2,0.957 |
|  |  |  |  |  |  |  |
| **PG** |  |  |  |  |  |  |
|  | **Flowering time** | **Final height** | **Branch number** | **Early height** | **Female fitness** | **Male fitness** |
| **Flowering time** | 0.269,1.276 | -0.718,0.213 | -0.689,0.234 | -0.552,0.186 | -0.345,0.034 | -0.587,0.143 |
| **Final height** | -0.718,0.213 | 0.318,1.549 | -0.184,0.85 | -0.229,0.598 | -0.061,0.353 | -0.121,0.704 |
| **Branch number** | -0.689,0.234 | -0.184,0.85 | 0.319,1.583 | -0.211,0.614 | -0.076,0.336 | -0.049,0.817 |
| **Early height** | -0.552,0.186 | -0.229,0.598 | -0.211,0.614 | 0.197,0.912 | -0.102,0.219 | -0.137,0.503 |
| **Female fitness** | -0.345,0.034 | -0.061,0.353 | -0.076,0.336 | -0.102,0.219 | 0.049,0.245 | -0.021,0.32 |
| **Male fitness** | -0.587,0.143 | -0.121,0.704 | -0.049,0.817 | -0.137,0.503 | -0.021,0.32 | 0.192,0.968 |
